# Active surveillance and contact precautions for preventing MRSA healthcare-associated infections during the COVID-19 pandemic

**DOI:** 10.1017/ash.2023.398

**Published:** 2023-09-29

**Authors:** Brian McCauley, Martin Evans, Loretta Simbartl, Makoto Jones, Gary Roselle, Anthony Harris, Eli Perencevich, Michael Rubin, Stephen Kralovic, Linda Flarida, Natalie Hicks

## Abstract

**Background:** Statistically significant decreases in methicillin-resistant *Staphylococcus aureus* (MRSA) healthcare-associated infections (HAIs) occurred in Veterans Health Administration (VA) facilities from 2007 to 2019 using active surveillance for facility admissions and contact precautions for patients colonized (CPC) or infected (CPI) with MRSA, but the value of these interventions is controversial. **Objective:** To determine the impact of active surveillance, CPC, and CPI on prevention MRSA HAIs, we conducted a prospective cohort study between July 2020 and June 2022 in all 123 acute-care VA medical facilities. In April 2020, all facilities were given the option to suspend any combination of active surveillance, CPC, or CPI to free up laboratory resources for COVID-19 testing and conserve personal protective equipment. We measured MRSA HAIs (cases per 1,000 patient days) in intensive care units (ICUs) and non-ICUs by the infection control policy. **Results:** During the analysis period, there were 917,591 admissions, 5,225,174 patient days, and 568 MRSA HAIs. Only 20% of facilities continued all 3 MRSA infection control measures in July 2020, but this rate increased to 57% by June 2022. The MRSA HAI rate for all infection sites in non-ICUs was 0.07 (95% CI, 0.05–0.08) for facilities practicing active surveillance plus CPC plus CPI compared to 0.12 (95% CI, 0.08–0.19; *P* = .01) for those not practicing any of these strategies, and in ICUs the MRSA HAI rates were 0.20 (95% CI, 0.15–0.26) and 0.65 (95% CI, 0.41–0.98; *P* < .001) for the respective policies. Similar differences were seen when the analyses were restricted to MRSA bloodstream HAIs. Accounting for monthly COVID-19 admissions to facilities over the analysis period using a negative binomial regression model did not change the relationships between facility policy and MRSA HAI rates in the ICUs or non-ICUs. There was no statistically significant difference in monthly facility urinary catheter-associated infection rates, a nonequivalent dependent variable, in the categories during the analysis period in either ICUs or non-ICUs. **Conclusions:** In Veterans Affairs medical centers, there were fewer MRSA HAIs when facilities practiced active surveillance and contact precautions for colonized or infected patients during the COVID-19 pandemic. The effect was greater in ICUs than non-ICUs.

**Figure:**
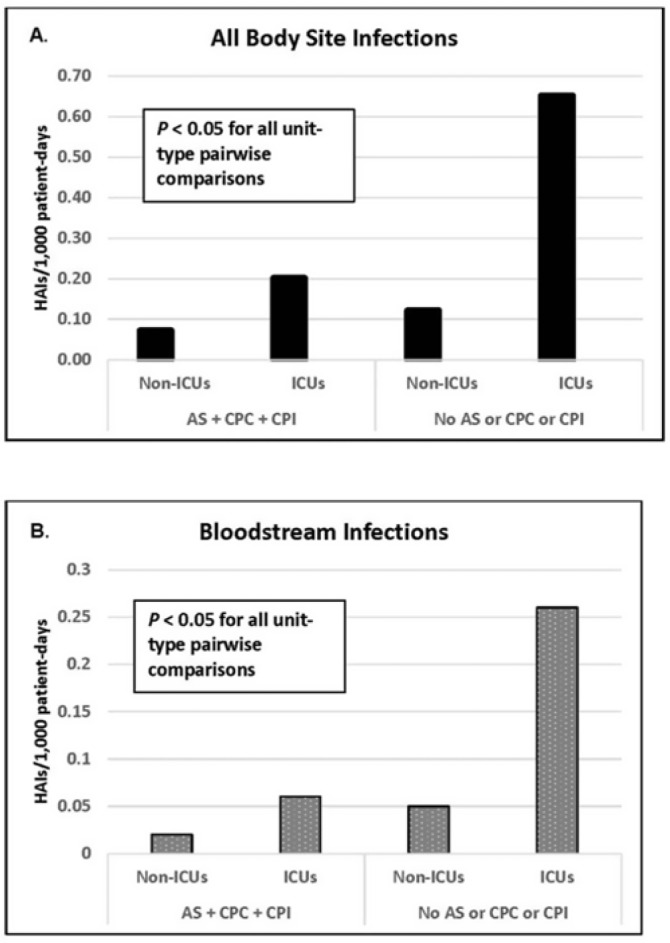

**Figure:**
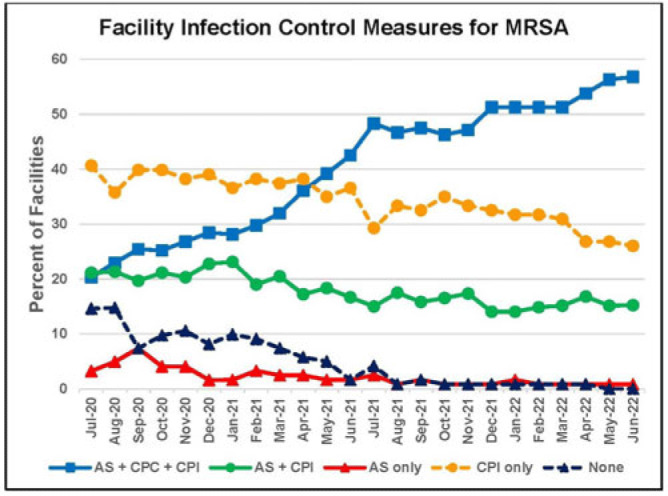

**Figure:**
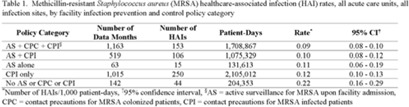

**Figure:**
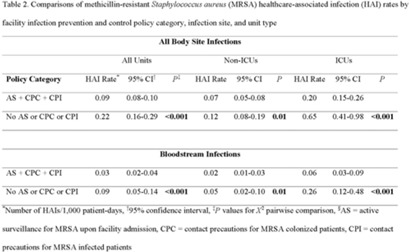

**Disclosures:** None

